# High-throughput computational screening and *in vitro* evaluation identifies 5-(4-oxo-4H-3,1-benzoxazin-2-yl)-2-[3-(4-oxo-4H-3,1-benzoxazin-2-yl) phenyl]-1H-isoindole-1,3(2H)-dione (C3), as a novel EGFR—HER2 dual inhibitor in gastric tumors

**DOI:** 10.32604/or.2023.043139

**Published:** 2023-12-28

**Authors:** MESFER AL SHAHRANI, REEM GAHTANI, MOHAMMAD ABOHASSAN, MOHAMMAD ALSHAHRANI, YASSER ALRAEY, AYED DERA, MOHAMMAD RAJEH ASIRI, PRASANNA RAJAGOPALAN

**Affiliations:** Department of Clinical Laboratory Sciences, College of Applied Medical Sciences, King Khalid University, Abha, Saudi Arabia

**Keywords:** Dual inhibitor, Drug discovery, EGFR/HER2 kinase, Gastric cancer, High-throughput screening

## Abstract

Gastric cancers are caused primarily due to the activation and amplification of the EGFR or HER2 kinases resulting in cell proliferation, adhesion, angiogenesis, and metastasis. Conventional therapies are ineffective due to the intra-tumoral heterogeneity and concomitant genetic mutations. Hence, dual inhibition strategies are recommended to increase potency and reduce cytotoxicity. In this study, we have conducted computational high-throughput screening of the ChemBridge library followed by *in vitro* assays and identified novel selective inhibitors that have a dual impediment of EGFR/HER2 kinase activities. Diversity-based High-throughput Virtual Screening (D-HTVS) was used to screen the whole ChemBridge small molecular library against EGFR and HER2. The atomistic molecular dynamic simulation was conducted to understand the dynamics and stability of the protein-ligand complexes. EGFR/HER2 kinase enzymes, KATOIII, and Snu-5 cells were used for *in vitro* validations. The atomistic Molecular Dynamics simulations followed by solvent-based Gibbs binding free energy calculation of top molecules, identified compound C3 (5-(4-oxo-4H-3,1-benzoxazin-2-yl)-2-[3-(4-oxo-4H-3,1-benzoxazin-2-yl) phenyl]-1H-isoindole-1,3(2H)-dione) to have a good affinity for both EGFR and HER2. The predicted compound, C3, was promising with better binding energy, good binding pose, and optimum interactions with the EGFR and HER2 residues. C3 inhibited EGFR and HER2 kinases with IC_50_ values of 37.24 and 45.83 nM, respectively. The GI_50_ values of C3 to inhibit KATOIII and Snu-5 cells were 84.76 and 48.26 nM, respectively. Based on these findings, we conclude that the identified compound C3 showed a conceivable dual inhibitory activity on EGFR/HER2 kinase, and therefore can be considered as a plausible lead-like molecule for treating gastric cancers with minimal side effects, though testing in higher models with pharmacokinetic approach is required.

## Introduction

Frequent amplifications of tyrosine kinase receptors (RTK), and the RTK-related gene plays a central role in the tumor development, progression, and proliferation in many solid tumors, including gastric cancers. Chromosomal instability and somatic mutations results in constitutive activation of RTKs that leads to uncontrolled cell proliferation [1]. Other molecular mechanisms leading to gastric tumors include the activation of RTKs, especially epidermal growth factor receptor (EGFR) (also known as HER1/ErbB1) by *Helicobacter pylori* (*H. pylori*) [2]. The most extensively studied RTKs in gastric cancer correspond to the human epidermal growth factor receptor family (ErbB). The ERbB family of receptor tyrosine kinases consists of the epidermal growth factor receptor (EGFR) (also known as HER1/ErbB1), human EGFR 2 (HER2/neu)/ERbB2, HER3/ErbB3 and HER4/ErbB4, and has a role in the tumorigenesis of many types of solid tumors, by promoting tumor angiogenesis and metastasis [1,3]. EGFR and HER2 are the most recognized tyrosine kinase receptors in gastric cancer [4], while overexpression of other recognized tyrosine kinase receptors in gastric cancers includes fibroblast growth factor receptor 2 (FGFR2) and MET [5,6]. The EGFR and HER 2, which are of the same family (HER/EGFR/ERBB), share a high degree of structural and functional homology and play an important role in pathogenesis of several cancers via cell proliferation, survival, migration, adhesion, differentiation, angiogenesis, invasion, and metastasis [7]. EGFR overexpression, and/or constitutive activation, has been shown to play an important role in the development and progression of certain aggressive types of solid tumors [8]. The reason behind EGFR high expression in a variety of tumors involves epigenetic causes, including transcriptional activation, gene amplification, oncogenic viruses, etc. [2,8]. Activation of the EGFR and/or its cognate ligands signaling network results in inhibition of apoptosis, cell migration and invasion, cellular differentiation, and transformation [9,10]. HER2 is another RTK, found in a variety of tumors and some of these tumors carry point mutations in the sequence specifying the transmembrane domain of HER2 [11]. The HER2 receptor is a transmembrane glycoprotein receptor with intracellular tyrosine kinase activity. EGFR and HER2 which are cell surface receptor tyrosine kinases (TKs), transduce growth signals through dimerization with HER family receptors [12]. The heterodimerization of EGFR with HER2 induces a more potent activation of EGFR, resulting in the autophosphorylation of tyrosine residues within the cytoplasmic domain of the receptors, and initiates a variety of signaling pathways including RAS/RAF/MEK/ERK/AKT/mTOR leading to cell proliferation and tumorigenesis [13]. In general, EGFR activates RAS which in turn activates RAF protein. Activation of RAF protein leads to activation of MEK kinase. Activated MEK kinase further activates ERK by phosphorylation. Activated ERK enters the nucleus to activate the transcription factor for cell proliferation, signifying the importance of EGFR activation in cell proliferation [14]. The overexpression of HER2 in gastric cancer is found mainly in the gastroesophageal junction and proximal parts compared to the distal parts of the stomach, in a heterogeneous pattern, and this overexpression and amplification is closely related to increased malignancy and poor prognosis. The preclinical and clinical trials of cancer therapy failed to produce consistent results due to the heterogeneity of gastric cancer, using ErbB receptor-targeted drugs. The use of IgG2 monoclonal antibody targeting EGFR was also studied in randomized trials [15]. Nevertheless, the results are not satisfactory, which could be attributed to non-standardized test quality for established biomarkers to evaluate the biological targets and also the intratumoral heterogeneity and concurrent genomic alterations in downstream molecules or other signaling pathways [16]. These hiccups were suggested as possible resistance mechanisms to EGFR-targeted therapies and hence the correlation between EGFR-HER2 and gastric cancer prognosis is widely studied to be used as a biomarker for a targeted and tailored therapy [17]. Dual inhibition strategies targeting both EGFR and HER2 have produced encouraging and promising outcomes and therefore is crucial to investigate them for benefiting patients with gastric cancers [18]. In this study, we have used high throughput computational screening and *in vitro* evaluations to short list candidates for further evaluations against cancer. The outcome of this approach will through light on the identified drug candidate and its analogues to be developed as novel therapeutics to fight gastric cancer.

## Materials and Methods

### Materials

Reagents and chemicals were purchased from Sigma Aldrich (St. Louis, MO, USA). EGFR (T790M/L858R) Kinase Assay Kit (Catalog # 40322) and HER2 Kinase Assay Kit (Catalog # 40721) were purchased from BPS Bioscience, SD, USA. KATO III and SNU-5 cell lines were purchased from ATCC (American Type Culture Collection, Rockville, MD, USA).

### Methods

### Three-dimensional structure retrieval and pre-processing

The three-dimensional structures of EGFR, and HER2 kinases with PDB-IDs 4HJO (EGFR bound to erlotinib), 4I23 (EGFR bound to dacomitinib), 3RCD (HER2 bound to TAK-285) were retrieved from the PDB database, www.rcsb.org. Crystal structures were selected based on three criteria, viz., maximum sequence coverage possible with minimum gaps, X-ray resolution, and the presence of a bound complex of standard known ligands. Retrieved EGFR structures resolutions were <2.80 Å, and HER2 structure was 3.2 Å. Before docking, target protein structures were processed by removing crystal waters, and adding polar hydrogens, using BIOVIA Discovery Studio Visualizer. For docking grid box generation, reference ligands bound to the respective structures were used. Docking results with protein-ligand complexes were visualized and analyzed in BIOVIA Discovery Studio Visualizer.

### Diversity based high-throughput virtual screening (D-HTVS)

For screening ChemBridge compounds, we used the diversity-based high-throughput docking technique developed by SiBioLead (LLP). Unlike the conventional high-throughput screening where each molecule in the entire database will be considered for docking, D-HTVS first screens the diverse set of molecules (scaffolds) from the ChemBridge database, against the target protein [19,20]. Based on the docking scores, the top 10 scaffolds/diverse molecules are selected, and all the structurally related molecules, having a tanimoto score of >0.6, for the top 10 scaffolds are retrieved from the pre-built ChemBridge database and docked (stage II docking) to the target protein. SiBioLead uses the Autodock-vina algorithm for all the docking calculations and these calculations for the D-HTVS method were performed in high-throughput mode, i.e., vina exhaustiveness of 1. Results of the stage II docking were parsed and ranked based on the docking energies. For this docking study, we used ChemBridge molecules having a molecular weight (MW) of >350 to <750.

### Conventional protein-ligand docking

Since the D-HTVS method runs on a high-throughput mode, for confirming the predictions from D-HTVS, a thorough docking using autodock-vina standard exhaustive mode was performed using the SiBDOCK module from SiBioLead server. Each docking produced 5 low-energy binding modes, and thus a binding pose with the lowest binding energy was considered for further analysis.

### Molecular dynamics simulations

GROMACS simulation package from WebGRO (www.simlab.edu) was used for simulating protein-ligand complexes according to previously published work [21]. In brief, before simulation, the protein-ligand complex was immersed in a triclinic box containing Simple Point Charge (SPC) water as a solvent model. The simulation system was typed with all-atom optimized potentials for liquid simulations (OPLS/AA) forcefield, which is a widely used forcefield for simulating biomolecules. Simulation system was added with NaCl as counter-ions, and further a 0.15 M NaCl was added to mimic the physiological condition. The whole simulation system was energy minimized for 5000 steps using the Steepest Descent method. Before the simulation, the system was equilibrated using NVT/NPT ensembles, viz., constant-number (N), constant-volume (V), constant-temperature (T), and constant-number (N), constant-pressure (P), constant-temperature (T) method. MD run was conducted for 100 ns using leap-frog integrator. Simulation trajectories were analyzed using Gromacs in-built trajectory analysis tool.

### Binding free energy calculations

In order to understand the binding free energy (ΔG binding) of an inhibitor with protein in an aqueous medium, GROMACS-based Molecular Mechanics Poisson-Boltzmann Surface Area (MM-PBSA) was used [22,23]. The binding free energy was estimated using the GROMACS utility g_mmpbsa. For computing ΔG binding, trajectory frames from the last 30 ns of the 100 ns simulation were used [22]. Results were analyzed and plotted using MmPbSaStat.py, and MmPbSaDecomp.py utilities.

### Enzyme inhibition assays

The EGFR [24] and Her2 kinase [25] assays were performed using the luminescence-based EGFR or HER2 Kinase assay kits from BPS Bioscience as per the manufacturer’s instructions. Briefly, to the master mix containing 1X Kinase Buffer, 500 µM ATP, and PTK substrate Poly (Glu: Tyr 4:1) in water, desired concentrations of the compound were added in a 96-well plate along with suitable blanks. The enzyme reaction was initiated by adding diluted EGFR(T790M/858R) or HER2 enzymes to the wells followed by incubation at 30°C for 45 min. Following this, Kinase-Glo Max reagent was added to each well, the plate was covered with aluminum foil and further incubated at room temperature for 15 min. The luminescence was measured using the BMG Omega microplate reader, with gain adjusted to optimal signals. IC_50_ values for the enzyme inhibition were calculated with GraphPad prism software. Staurosporine was used as a positive control as recommended by the manufacturer.

### Cell culture and anti-proliferation assays

KATO III and SNU-5 cells were cultured in ATCC-formulated Iscove’s Modified Dulbecco’s Medium (Catalog No. 30-2005) with 20% FBS, antibiotics as per standard protocols. MTT assay was performed when cells reached 80% confluency using the protocol of previously published work [26]. Percentage cell proliferation inhibition was calculated and GI_50_ (half dose for Growth inhibition) was presented with GraphPad Prism 6.0 software.

## Results

### High-throughput virtual screening of ChemBridge library identify top EGFR binding compounds

**Figure 1:**
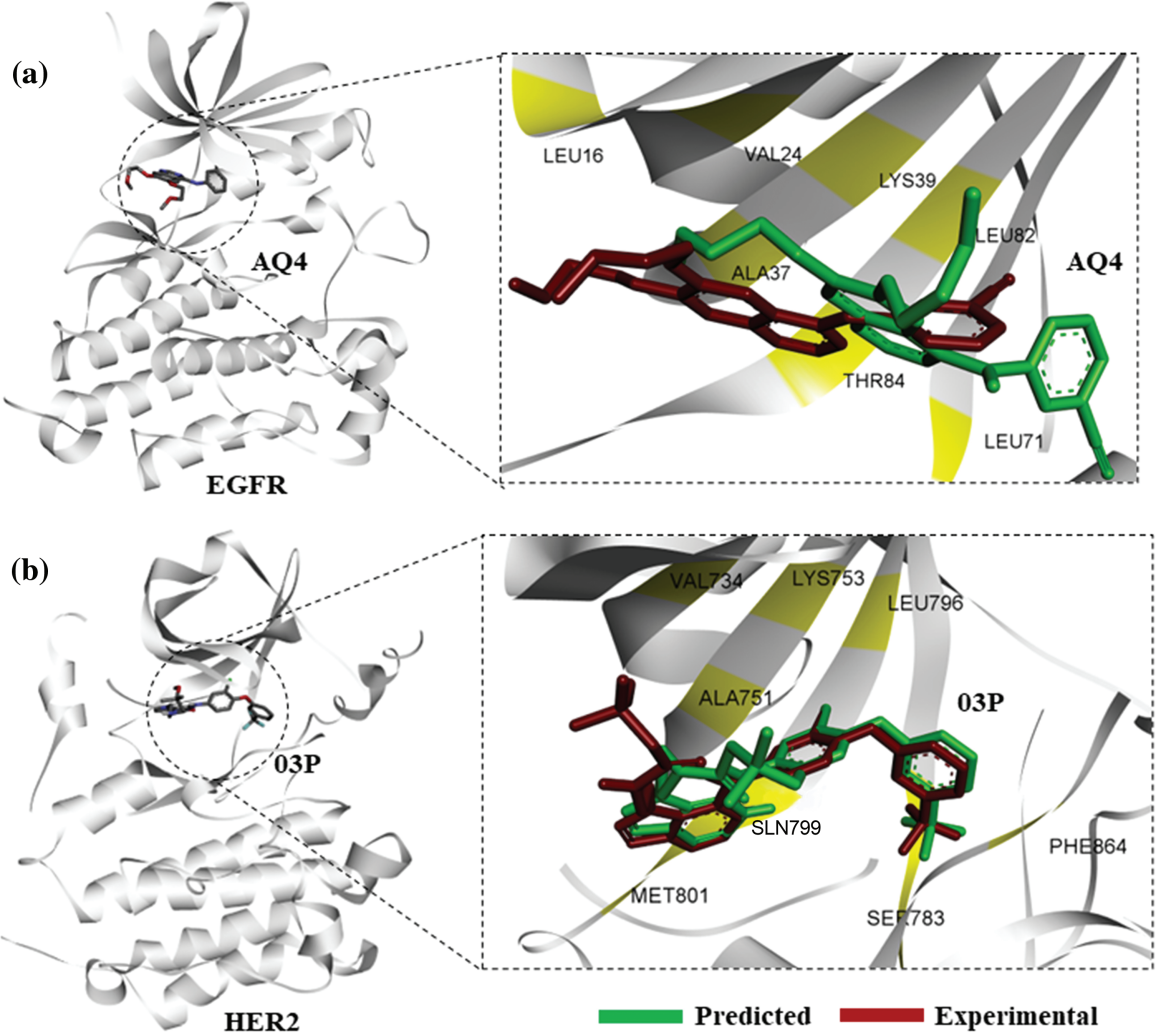
Protein-ligand docking of known kinase inhibitors. (a) Predicted binding pose of AQ4 (erlotinib) to EGFR (green), in comparison with experimental binding pose from 4hjo (red). (b) Binding pose prediction based on protein-ligand docking of 03P with HER2 kinase (3rcd, green), in comparison with experimentally derived binding pose (red).

To identify novel small molecules preferentially binding to the active conformations of EGFR-TKD, and HER2 kinase, we used computational high-throughput docking approach. To first establish the docking protocol that would be used for screening large combinatorial chemical libraries, we performed computational docking of known inhibitors of EGFR, and HER2, namely erlotinib (AQ4), and 03P (TAK-285), respectively, with EGFR, and HER2 kinases in their active conformations. Docking of erlotinib, and 03P closely reproduced the binding modes derived experimentally in PDB entries for EGFR, 4hjo, as well as for HER2, 3rcd [27] (Fig. 1).

**Figure 2:**
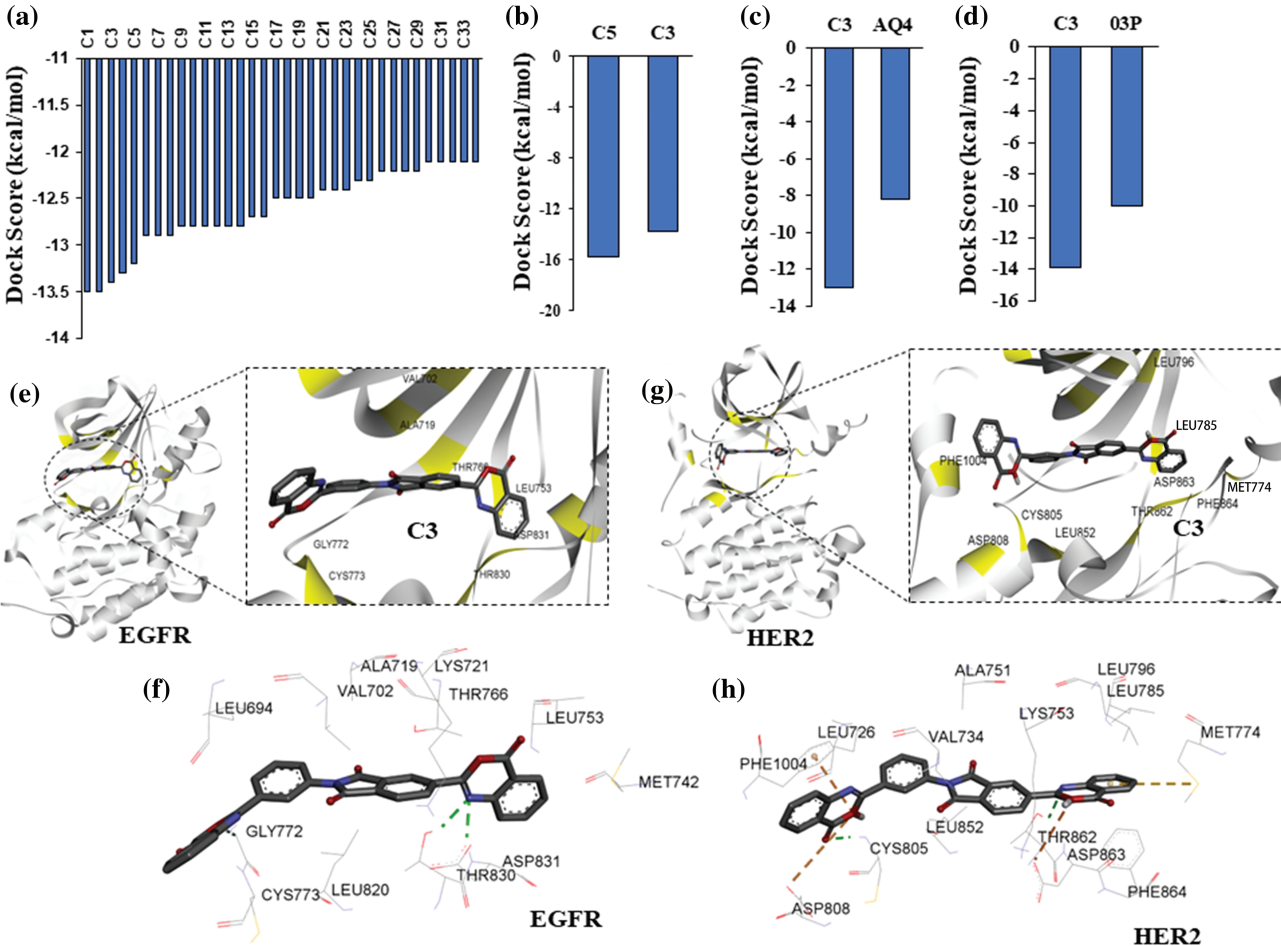
High-throughput virtual screening identify targeted compounds for EGFR and HER2. (a) Predicted binding scores for the ChemBridge compounds against EGFR kinase. Histogram includes compounds having docking score >2 standard deviation from the mean docking scores (−9.7 kcal/mol). (b) Docking scores, >2 standard deviation from the mean, for top EGFR compounds from (a) screened against HER2 kinase. (c) Predicted docking score for compound C3 and a known inhibitor AQ4 against EGFR. (d) Predicted docking score for compound C3 and a known inhibitor 03P against HER2. (e) Interacting residues between compound C3 and EGFR. (f) 2D representation of compound C3 interactions at the kinase site of EGFR. (g) Interacting residues between compound C3 and HER2. (h) 2D representation of compound C3 interactions at the kinase site of HER2.

Structural superimposition of experimentally derived binding conformation with our predicted binding mode resembles a similar binding manner. Binding residue analysis shows both erlotinib, and 03P interact with crucial residues at the EGFR, and HER2 kinase domains respectively (Fig. 1) [28]. We then proceeded with a high-throughput virtual screening of the ChemBridge library. To predict novel drug-like compounds with increased potency and less cytotoxicity, we virtually screened compounds from the ChemBridge library having molecular weights >350 and <750. Erlotinib and 03P were used as reference ligands for EGFR and HER2 respectively for defining the docking grid box for our virtual screening process. This ensures that the docking grid box covers the entire active site of the EGFR and HER2 protein. Using previously established diversity-based high-throughput virtual screen technology (D-HTVS), around 0.75 million compounds were screened from the ChemBridge database. Autodock-vina software from SiBioLead LLP was used for the high-throughput virtual screening [29]. D-HTVS of ChemBridge library identified top candidates having high binding affinity for EGFR at the kinase site in its active conformation. A total of 1185 compounds out of 0.75 million were predicted to bind to EGFR kinase with low −ve docking energies. Docking energy for the top-most compound was −13.5 kcal/mol, and for the least compound was −6.5 kcal/mol. To rank top compounds with high confidence for further analyses, we selected compounds having docking scores that are two standard deviations (95% confidence) from the mean docking energy for all the identified compounds (Fig. 2a). This threshold yielded 35 compounds having docking scores above −12.0 kcal/mol for the EGFR kinase (Fig. 2a).

### Protein-ligand docking of HER2 kinase predicts dual inhibitors of EGFR and HER2

In order to identify dual inhibitors having preferential binding for both EGFR and HER2 kinases, we performed computational docking of ChemBridge compounds identified from our initial high-throughput screening with EGFR kinase against the active site of HER2 kinase (Fig. 2a). Similar to EGFR screening, we screened the predicted top HER2 binding compounds by two standard deviations away from the mean (Fig. 2a). With this threshold, two compounds (C5 and C3) out of 1185 were identified to have preferential binding for both EGFR and HER2 kinases (Fig. 2b). Based on the predicted docking scores, compound C3,5-(4-oxo-4H-3,1-benzoxazin-2-yl)-2-[3-(4-oxo-4H-3,1-benzoxazin-2-yl)phenyl]-1H-isoindole-1,3(2H)-dione, had an almost equal binding affinity (preference) for both EGFR and HER2 (Figs. 2a and 2b), therefore, compound C3 was chosen for further analysis. We then compared the docking affinity of the predicted compound C3 with the known EGFR and HER2 inhibitors. Protein-ligand docking in high precision/exhaustive mode indicates that predicted compound C3 binds with better binding energy to EGFR (−13.0 kcal/mol) and HER2 (−13.9 kcal/mol) compared to their respective known inhibitors, viz., AQ4, EGFR inhibitor (−8.2 kcal/mol), and 03P, HER2 inhibitor (−10.0 kcal/mol) (Figs. 2c and 2d). Binding pose analysis of compound C3 with EGFR indicates the predicted compound to form hydrogen bonds with Cys773, The 830, and Asp 831 (Fig. 2e). Additionally, compound C3 formed hydrophobic interactions with EGFR residues, Leu694, Val702, Met742, Leu753, Thr766, Asp776, Leu820, and Phe832 (Fig. 2f), contributing to the observed binding energies. Analysis of compound C3 binding to HER2 kinase indicates that C3 forms a total of three hydrogen bonds with HER2 residues Cys805, Thr862, and Asp863 (Fig. 2g). The compound C3 also formed one salt-bridge interaction with Lys753 and hydrophobic interactions with residues Leu726. Lys753, Leu785, Leu796, Leu800, Phe864, and Phe1004 at the active site of HER2 (Fig. 2h).

### Molecular dynamics simulation of EGFR-C3 complex

**Figure 3:**
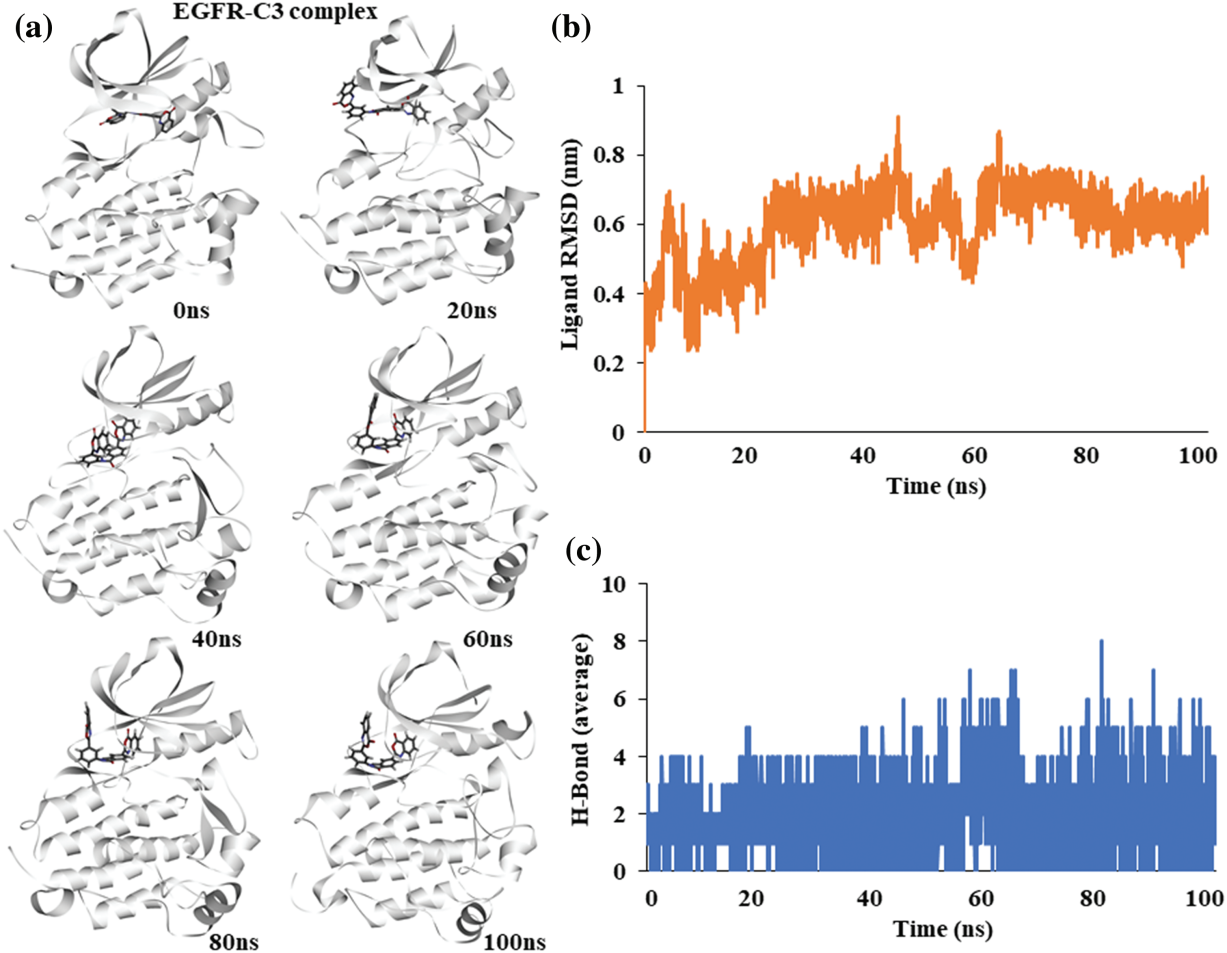
Molecular dynamics simulation of the EGFR-C3 complex (a) Snapshot of trajectories taken at different time points (b) Predicted ligand root mean square deviation of compound C3 (c) Predicted average hydrogen bonds between compound C3 and EGFR for 100 ns a simulation.

To assess the stability of compound C3 binding to EGFR in its active conformation, we performed fully solvated molecular dynamics simulation for 100 ns using WebGRO server available at https://simlab.uams.edu/ [21]. Analysis of the path of compound C3 shows that it stays bound to the active site of EGFR kinase. Snapshots of different simulation time points show a slight torsional rotation of compound C3 at the binding site; however, the binding region did not alter during the simulation, indicating the binding stability of compound C3 to EGFR (Fig. 3a).

The Root Mean Square Deviation (RMSD) of the ligand shows an increase at the start of the simulation and stabilizes after 50 ns, indicating a stable complex (Fig. 3b). The average number of hydrogen bonds between EGFR and compound C3 remained stable during the simulation; however, it increased toward the end of the simulation (Fig. 3c).

### Molecular dynamics simulation of HER2-C3 complex

**Figure 4:**
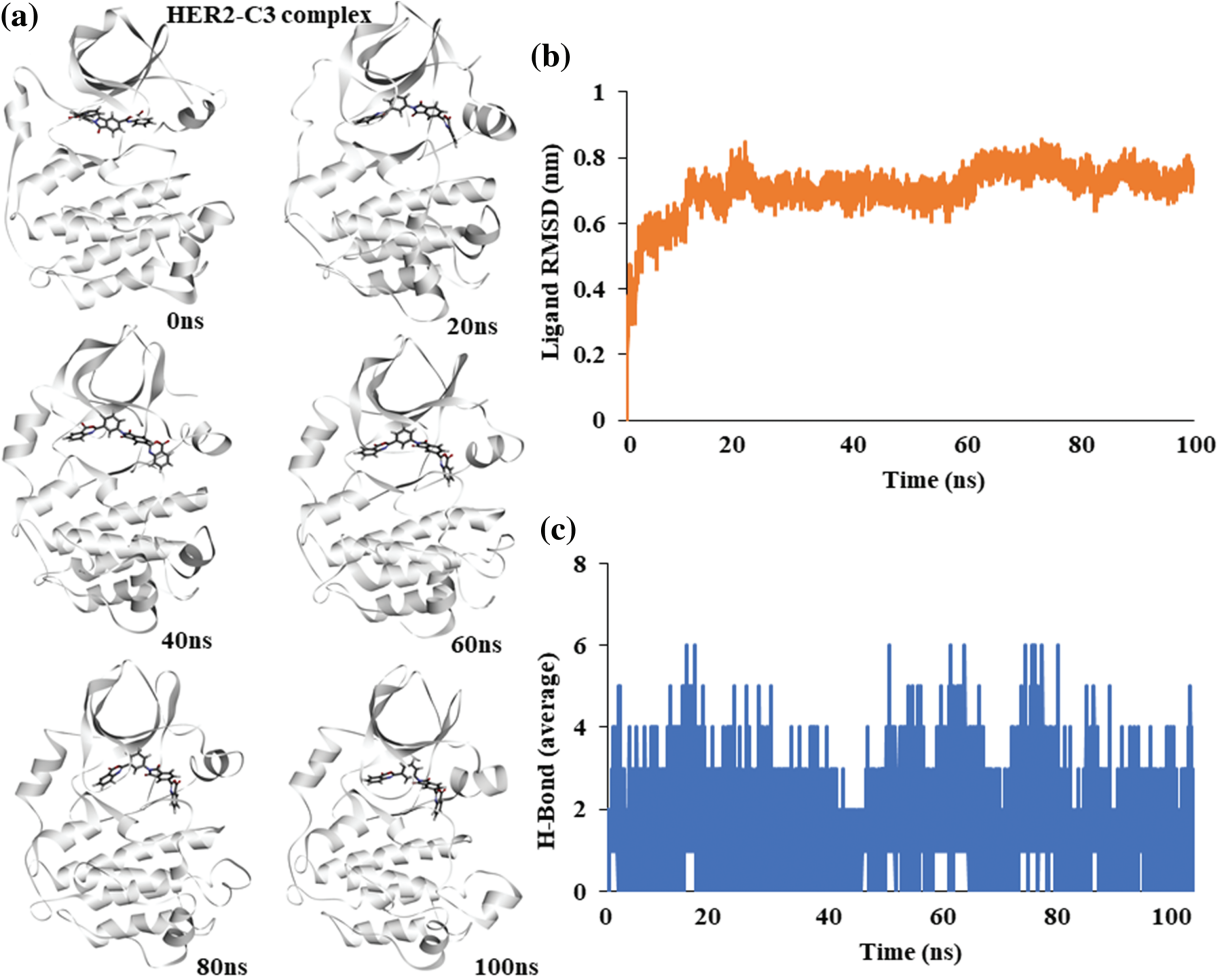
Molecular dynamics simulation of the HER2-C3 complex (a) Snapshot of trajectories taken at different time points during the simulation. (b) Predicted ligand root mean square deviation of compound C3 when bound to HER2. (c) Predicted average hydrogen bonds between compound C3 and HER2 for a 100 ns simulation.

Similar to EGFR, the binding stability of C3 with HER2 kinase was assessed. An atomic molecular dynamics simulation of the HER2-C3 complex for 100 ns showed a stable binding of C3 with HER2. Snapshots of simulation trajectories at different time points show no notable changes or deviation in C3 binding (Fig. 4a).

Ligand RMSD shows a stable binding of compound C3 to HER2 throughout the 100 ns simulation (Fig. 4b). Similarly, average hydrogen bonds between the ligand, C3, and the protein, HER2, remained stable throughout the simulation, suggesting a good fit (Fig. 4c).

### Solvent based binding free energy calculation of EGFR-C3, and HER2-C3 complex

**Figure 5:**
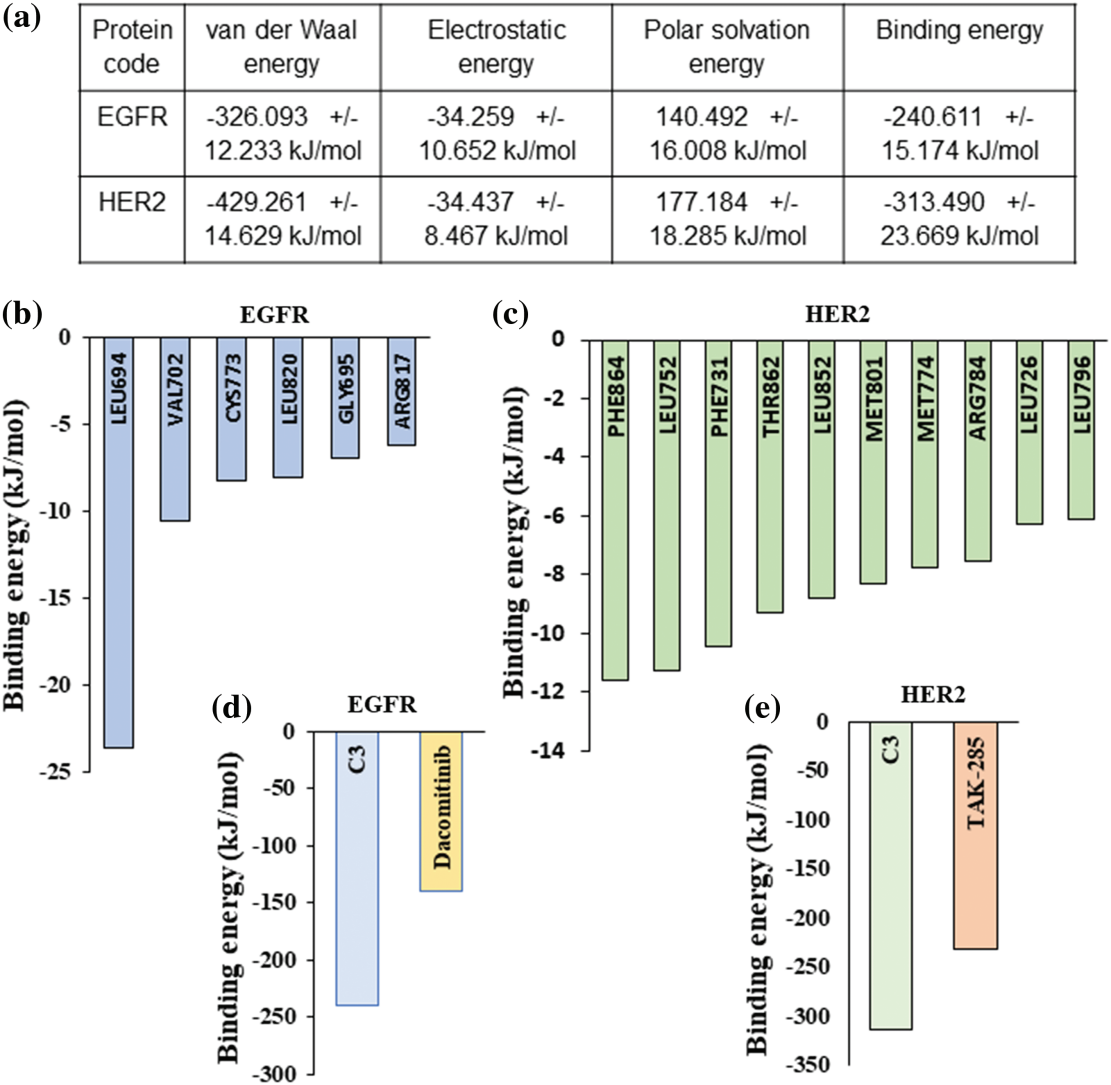
MM/PBSA-based binding free energy predictions for compound C3 (a) Predicted binding free energy (ΔG_binding_) for compound C3 to EGFR and HER2 calculated based on MM/PBSA scoring from simulation trajectories (total cutoff value: −63.59 kJ/mol for EGFR and −87.42 kJ/mol for HER2). (b) Residue level binding energy contribution for the EGFR-C3 complex (cut off value −6 kJ/mol). (c) Residue level binding energy contribution for the HER2-C3 complex (cutoff value −6 kJ/mol). (d) MD simulation and MM/PBSA-based predicted binding free energy comparison between compound C3 and dacomitinib bound to EGFR (e) MD simulation and MM/PBSA-based predicted binding free energy comparison between compound C3 and dacomitinib bound to HER2.

For calculating the binding free energy (deltaGbind) of EGFR-C3 and HER2-C3 complexes in an aqueous solvent, we performed the Molecular Mechanics Poisson-Boltzmann Surface Area (MM-PBSA) approach using the Gromacs simulation package [22,23]. The last 20 ns frames from the 100 ns MD simulation were used to calculate the binding free energy for the EGFR-C3 and HER2-C3 complexes. Results indicate compound C3 shows significant binding free energy for EGFR and HER2 kinases. Compound C3 with EGFR shows a binding free energy of −240.611 kJ/mol or −57.05 kcal/mol (Fig. 5a). Similarly, the binding free energy of compound C3 with HER2 was predicted to be −313.49 kJ/mol or −74.9 kcal/mol (Fig. 5a).

Analyzing the residue-based contribution for the predicted binding energy indicates that Leu694, Val702, Cys773, Leu820, Gly695, and Arg817 residues to have greater contributions in the EGFR-C3 complex (Fig. 5b). Likewise, HER2 residues Phe864, Leu752, Phe731, Thr862, Leu852, Met801, Met774, Arg784, Leu726, and Leu796 provide major contributions for the HER2-C3 binding (Fig. 5c). For assessing the predicted binding affinity of the compound C3 to other known inhibitors of EGFR and HER2, we performed MM-GBSA analysis of dacomitinib, and TAK-285 bound to EGFR and HER2, respectively. The predicted binding free energy of dacomitinib to EGFR was −139.56 kJ/mol (Fig. 5d) and the predicted binding free energy of TAK-285 to HER2 was −232.56 kJ/mol (Fig. 5e), indicating that compound C3 has favorable binding to both EGFR and HER2.

### C3 inhibited EGFR and HER2 enzymes in vitro and controlled gastric cancer cell proliferations

**Figure 6:**
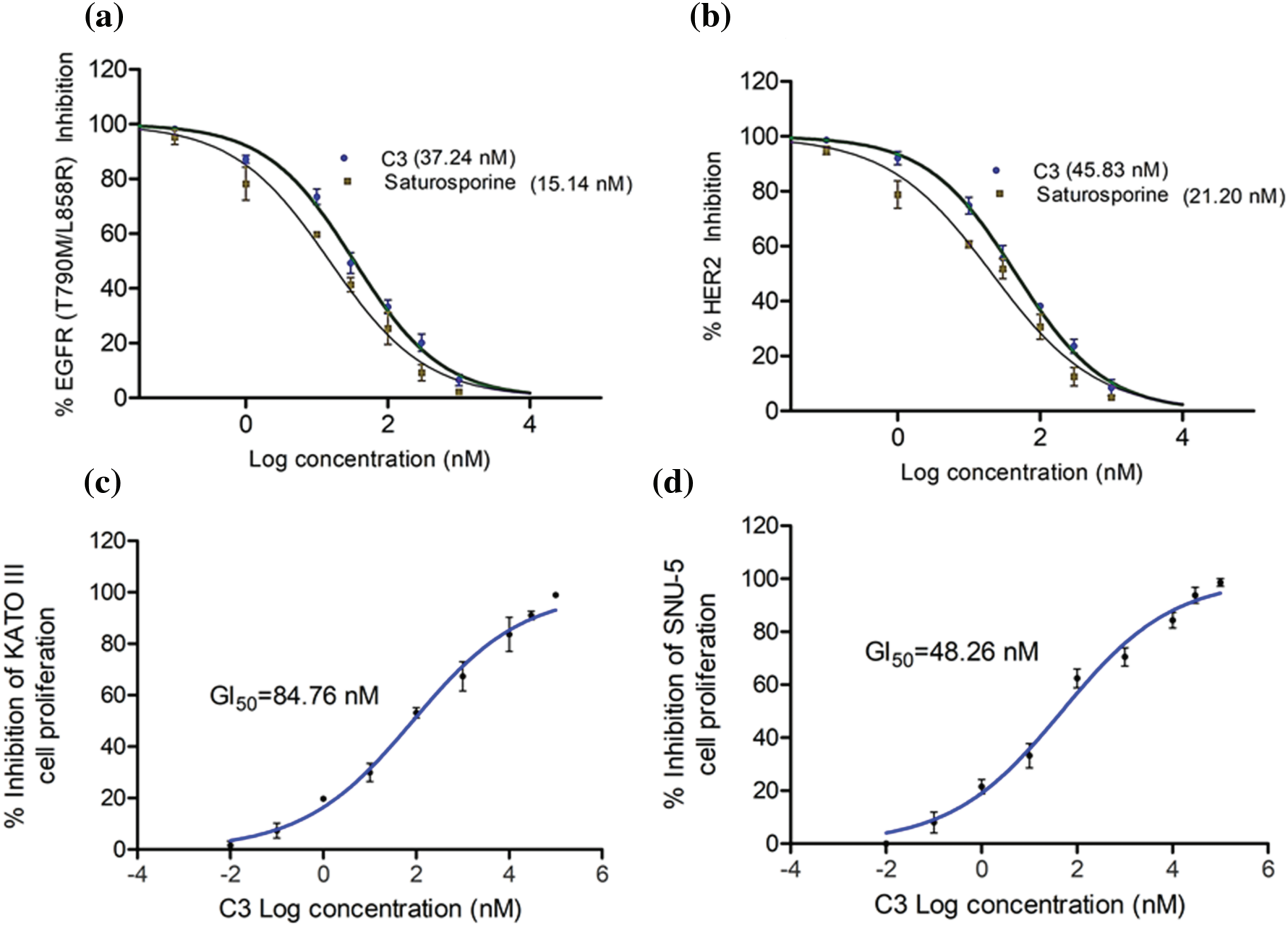
IC_50_ of C3 against (a) EGFR and (b) HER2 enzymes. GI_50_ of C3 in controlling proliferation of (c) KATO III and (d) SNU-5 cells.

To verify the *in vitro* efficacy of the observed computational effects, we tested the compound for its inhibition of EGFR and HER2 enzymes. C3 effectively inhibited both EGFR and HER2 enzymes with respective IC_50_ values of 37.24 and 45.83 nM (Figs. 6a, 6b).

The reference compound Staurosporine inhibited EGFR and HER2 enzymes with IC_50_ values of 15.14 and 21.20 nM, respectively (Figs. 6a, 6b). In order to evaluate if the enzyme inhibitory effects of C3 are translated into the anticancer efficacy, we carried out the antiproliferative assay with the compound in the gastric cancer KATO III and SNU-5 cells. As observed in Figs. 6c and 6d, the compound inhibited the proliferation of both gastric carcinoma cells. The GI_50_ values were found to be 84.76 and 48.26 nM in KATO III and SNU-5 cells, respectively.

## Discussion

High-throughput virtual screening of the ChemBridge library identified small molecules binding to EGFR, and subsequent docking of top compounds against HER2 kinase identified compound C3, 5-(4-oxo-4H-3,1-benzoxazin-2-yl)-2-[3-(4-oxo-4H-3,1-benzoxazin-2-yl) phenyl]-1H-isoindole-1,3(2H)-dione, that has preferential binding for both EGFR and HER2 at their active conformation. Structural analysis of experimental models and protein-ligand docking of known inhibitors of EGFR and HER2 identified key residues/regions necessary for inhibiting the kinase activity of EGFR and HER2 [27,28,30]. Protein-ligand interaction analysis of predicted ChemBridge compounds showed the top compounds with high binding affinity targeting the key residues, including The830, and Asp831 in EGFR and Cys805, Thr862, and Asp863 in HER2. These residues were previously known to have an impact on kinase activity [27,28].

Apart from conventional protein-ligand docking, in this work, we used a fully solvated atomistic molecular dynamics simulation of the top predicted compound, C3, with EGFR and HER2. Since MD simulations were performed close to physiological conditions, i.e., with water and salt ions, predictions from such MD simulations help to understand the stability of the protein-drug complex. Our simulations clearly indicate that compound C3 has significant binding stability to both EGFR and HER2.

To back up the prediction even more, we used MM-PBSA to look at the MD paths of EGFR- and HER2-C3 complexes Solvent-based binding energy predictions appear to mimic experimental enzyme inhibition because binding energies predicted from such studies are calculated by accounting for solvents in the system that closely match physiological conditions [22,23]. Predicted binding energy values clearly show that the predicted compound C3 has a superior binding affinity compared to known inhibitors of EGFR and HER2. Experimental validation of compound C3 corroborates with our predictions. The *in vitro* kinase inhibition assay clearly demonstrates that compound C3 inhibits both EGFR and HER2 in a dose-dependent manner. The identified IC_50_ of compound C3 for both EGFR and HER2 is in the nanomolar range and is comparable to the known inhibitor Staurosporine [31]. Similarly, treatment of compound C3 in KATO III and SNU-5 cells showed significant inhibition in cell proliferation, especially in a nanomolar range, suggesting drug-like properties of compound C3.

## Conclusion

Using a combination of high-throughput virtual screening and *in vitro* analysis, we found that compound C3 from the ChemBridge small molecule library is a lead candidate that selectively targets EGFR and HER2 to stop cell growth in cell culture models of gastric cancer. In this work, our approach was based on a computational high-throughput screen followed by *in vitro* analysis to identified compound C3, which binds preferentially to EGFR and HER2. Since most of the ChemBridge compounds have good drug-like properties, we hypothesize that with further evaluations in higher models, C3 could be developed as a selective dual inhibitor of EGFR/HER2 kinase as a treatment regimen for solid tumors, especially gastric tumors.

## Data Availability

The datasets generated during and/or analyzed during the current study are available from the corresponding author on reasonable request.
